# Recently Delisted Songbird Harbors Extensive Genomic Evidence of Inbreeding, Potentially Complicating Future Recovery

**DOI:** 10.1111/eva.70052

**Published:** 2024-12-09

**Authors:** Anna María Calderón, Andrew W. Wood, Zachary A. Szpiech, David P. L. Toews

**Affiliations:** ^1^ Department of Biology, 619 Mueller Laboratory Pennsylvania State University University Park Pennsylvania USA

**Keywords:** conservation genomics, genetic load, inbreeding, runs of homozygosity, *Setophaga kirtlandii*

## Abstract

The Kirtland's warbler ( *Setophaga kirtlandii* ) is a rare migratory passerine species and habitat specialist of the North American Jack Pine Forests. Their near extinction in the 1970s classified them as endangered and protected under the Endangered Species Act of 1973. After decades of intense conservation management, their population size recovered, and they were delisted from federal protection in 2019. We explore the genomic consequences of this harsh bottleneck and recovery by comparing the genomic architecture of two closely related species whose population sizes have remained large and stable, Hooded Warblers ( *Setophaga citrina* ) and American Redstarts ( *Setophaga ruticilla* ). We used whole‐genome sequencing to characterize the distribution of runs of homozygosity and deleterious genetic variation. We find evidence that Kirtland's warblers exhibit genetic patterns consistent with recent inbreeding. Our results also show that Kirtland's warblers carry an excess proportion of deleterious variation, which could complicate management for this conservation‐reliant species. This analysis provides a genetically informed perspective that should be thoroughly considered when delisting other species from federal protections. Through the increasing accessibility of genome sequencing technology, it will be more feasible to monitor the genetic landscape of recovering populations to ensure their long‐term survival independent of conservation intervention.

## 1 Introduction

The application of genomic tools to the conservation of wildlife has had profound implications for the ability of conservation practitioners to maintain and increase populations of endangered taxa. Most species of conservation concern have low population sizes that can result in inbreeding. For many years, researchers have documented that inbreeding between closely related individuals generally results in a fitness loss for the resulting offspring (Charlesworth and Willis 2009; Crnokrak and Roff 1999; Hedrick and Garcia‐Dorado 2016; Huisman et al. 2016; Keller and Waller 2002; Wright, Tregenza, and Hosken 2008). Historically, the tools of conservation genetics only allowed researchers to measure the extent of inbreeding by focusing on single genome‐wide metrics like the inbreeding coefficient, though accurate estimates have been difficult to obtain without a comprehensive pedigree (Kardos, Luikart, and Allendorf 2015; Kardos et al. 2016; Wang 2016). Advancements in sequencing technology allow us to directly quantify inbreeding through runs of homozygosity (ROH), contiguous tracts of homozygotes, the result of long haplotypes inherited identical by a descent from a recent common ancestor (Ceballos et al. 2018). This can lower fitness by increasing the exposure of genetic load, that is, the realized load, specifically by unmasking recessive deleterious variants and by increasing the frequency of rare recessive alleles (Szpiech et al. 2013). While inbreeding promotes the purging of strongly damaging variants (Keller and Waller 2002), weak to moderately damaging variants are purged less efficiently and can drift to fixation (Leon‐Apodaca et al. 2024; Robinson et al. 2016). A careful consideration of the realized load is particularly important to the conservation of small populations as the additive effects of many deleterious variants may lower fitness and increase the risk of extinction (Kyriazis, Wayne, and Lohmueller 2021; Schiegg et al. 2006; Stoffel et al. 2021).

Kirtland's warblers ( *Setophaga kirtlandii* ) are one of the rarest songbirds in North America. Their rarity is owed to their breeding habitat specificity in early successional Jack Pine forests, and they are heavily restricted to the northern Lower Peninsula of Michigan (Probst 1986). Landscape modifications including the suppression of large cyclical fires, the expansion of agriculture, and industrial tree farming led to a major loss of a suitable nesting habitat. These landscape changes also encouraged the range expansion of the brown‐headed cowbird ( *Molothrus ater* ), an obligate brood parasite native to North America. Cowbird chicks outgrow and outcompete their host‐siblings leading to poor nesting success in species that cannot recognize or remove parasitic eggs (Cooper, Rushing, and Marra 2019). While cowbirds are native to North America, empirical evidence suggests that they were not considered a common species in adjacent regions (e.g., southern Ontario and Ohio) until the mid‐1800s, possibly expanding into Michigan around the 1870s (Mayfield 1961; Probst 1986). These close interplaying factors played an important ecological role in the rapid decline of the Kirtland's warblers that led to their classification as an endangered species under the Endangered Species Act of 1973. Despite the implementation of aggressive management starting in 1972 (Fish and Wildlife Service 2019), populations remained stagnant for nearly two decades until managers began restoring critical jack pine forest habitat in the 1980s (Donner, Probst, and Ribic 2008). After decades of intense and focused management, Kirtland's warblers recovered and were delisted from the Endangered Species Act in 2019, marking a conservation success story as rare as the Kirtland's warblers themselves (Fish and Wildlife Service 2019). Although Kirtland's warblers are no longer federally protected, they are still a conservation‐reliant species and continue to be managed and monitored (Bocetti, Goble, and Scott 2012; Cooper, Rushing, and Marra 2019).

In addition to an increased chance of extinction, bottlenecks increase the likelihood of inbreeding and subsequent inbreeding depression, which is the reduced fitness associated with consanguineous mating (Becker et al. 2016; Hemmings, Slate, and Birkhead 2012; Kyriazis, Wayne, and Lohmueller 2021; Robinson et al. 2019; Sittmann, Abplanalp, and Fraser 1966). These factors were not considered thoroughly in the decision to delist the Kirtland's warblers (Fish and Wildlife Service 2019). Here, we use whole‐genome sequencing to explore the genomic underpinnings of strong bottlenecks in the Kirtland's warbler. We take advantage of the fact that their closest relatives, Hooded warblers ( *Setophaga citrina* ) and American redstarts ( *Setophaga ruticilla* ), have retained large, stable populations to draw comparisons and explore how their demographic histories influenced the prevalence of ROH and the distribution of deleterious variation.

## 2 Methods

### 2.1 Sampling and Sequencing

We used whole‐genome resequencing across multiple individuals, *Setophaga kirtlandii* (*n* = 7), and compared them to closely related warbler species with differing population histories: *Setophaga citrina* (*n* = 5) and *Setophaga ruticilla* (*n* = 7). Our samples included 14 individuals from a previous study (Baiz et al. 2021)—now sequenced to a higher coverage—as well as six new individuals that were wild caught using mist nets and playback songs and blood‐sampled from the brachial vein (USFS master banding permit #24043) (Table S1, Figure S1). DNA from new samples were extracted using 75 μL of blood following the Qiagen DNeasy Blood and Tissue Kit protocol. DNA libraries were then created with the Illumina TruSeq Nano kit, targeting 350‐bp insert sizes. Samples were indexed, and the libraries were pooled and sequenced with Illumina NextSeq 500. We included all individuals at once on a single lane run three times with paired‐end 150‐bp chemistry to target an overall coverage of 20×.

### 2.2 Variant Calling and Filtering

We aligned all whole‐genome reads to the chromosome‐level reference genome of the Myrtle Warbler ( *Setophaga coronata* ; PRJNA325157; [Baiz et al. 2021]). First, using AdapterRemoval V2.1.7 (Schubert, Lindgreen, and Orlando 2016), we trimmed and collapsed the overlapping paired reads with the following parameters: ‐‐collapse ‐‐trimns ‐‐minlength 20 ‐‐qualitybase 33. We aligned reads using BowTie2 V2.3.5.1 (Langmead and Salzberg 2012) with the “very‐sensitive‐local” presets and set the ‐X flag, the maximum gap length for valid paired‐end alignments, to 700 bp. After our reads were aligned, we converted the resulting SAM files into BAM files and sorted them with SAMTOOLS V1.18 (Danecek et al. 2021). Using PicardTools V2.20.8 (https://broadinstitute.github.io/picard/) we flagged any duplicated reads. Lastly, we indexed the resulting marked BAM files with SAMTOOLS.

We used the Genomic Analysis Tool Kit's (GATK V3.8) HaplotypeCaller (Poplin et al. 2018) to identify variants and determine per‐read likelihood haplotypes, using the ‐‐emitRefConfidence GVCF option. We then called variants across all GVCF's with the GATK tool “GenotypeGVCFs,” which generated a single joint variant database, then indexed, and implemented the following filters using BCFTOOLS V1.18 (Danecek et al. 2021). Sites with a combined read depth across individuals < 255 or > 401 were filtered out (these values correspond to the 5th and 95th percentiles for a total depth of our samples, respectively). Variants were further filtered based on site quality, keeping any variants with a quality score > 50. Multi‐nucleotide polymorphisms, indels, complex variants, and sites with more than one alternate allele were excluded. Because statistical tests for excess heterozygosity are underpowered for small sample sizes, we filtered for excess heterozygosity for each species separately. A similar approach was taken in Leon‐Apodaca et al. (2024), Robinson et al. (2018), and Wood et al. (2023). For *S. citrina* , with a sample size of 5, we excluded any sites that exceeded four or more sites of heterozygous genotypes. For *S. kirtlandii* and *S. ruticilla* , each with a sample size of 7, sites with excess heterozygosity were defined as sites with six or more heterozygotes and were excluded. Genotypes required a minimum of six and a maximum of 34 supporting reads, which were based on the 5th and 95th percentile values of all samples per species. Genotypes with quality scores < 20 were excluded, and we only included sites with < 3 missing genotypes. We filtered out unmapped scaffolds as well as the Z‐chromosome to limit any sex bias. We then recombined all VCFs and filtered out monomorphic sites from this joint vcf file. For some analyses, we also filtered monomorphic sites per species.

### 2.3 Population Structure and Relatedness

We explored population structure using allele sharing distance (asd) V1.1.0a (https://github.com/szpiech/asd), a distance‐based clustering metric of population stratification. ASD returned a dissimilarity matrix, which was then used for multidimensional scaling analysis (MSD) using the R function *cmdscale* and calculated Weir and Cockerman's Fst in vcftools using the ‐‐weir‐fst‐pop flag to show that there is no admixture between species (Figure S2A) and no within‐species population structure (Figure S2B–D). We implemented two kinship analysis to verify that our samples were not closely related, which can bias ROH calling. We used the program KING V2.3.2 (Manichaikul et al. 2010), an algorithm that infers relationships between individuals by modeling genetic distance from allele frequencies. This method is suitable for high‐throughput data like whole‐genome sequencing and is applicable to small populations and populations with high stratification or homogeneity. We also used ASD's “ibs” function to conduct an allele sharing analysis (Pemberton et al. 2010), which verified that none of our samples were closely related (Figure S3).

### 2.4 Runs of Homozygosity

Runs of homozygosity were called from genotype data with the program GARLIC (Szpiech, Blant, and Pemberton 2017). GARLIC implements a model‐based likelihood approach, considering genotype quality scores for identifying probable autozygosity regions and inferring runs of homozygosity. To run GARLIC, we specified the following parameters ‐‐auto‐winsize ‐‐auto‐overlap‐frac ‐‐winsize 100 ‐‐gl‐type GQ ‐‐resample 40 ‐‐size‐bounds 1000000 2000000 3000000 4000000 5000000. By manually setting the size‐bound thresholds, GARLIC categorized autozygous regions as ROH < 1 Mb, 1 Mb<ROH < 2 Mb, 2 Mb<ROH < 3 Mb, 3 Mb<ROH < 4 Mb, 4 Mb<ROH < 5 Mb, and ROH > 5 MB. We also manually filtered out any ROH < 0.5 Mb to avoid any false calls.

The lengths of ROH can be used to estimate the coalescence times of the underlying haplotypes. Coalescence times are proportional to effective population sizes and can illuminate the extent of inbreeding in the population over time. We aged haplotypes in ROH using the equation $l=\frac{100}{2g}$, where *l* is the length of the ROH segment in centimorgans and *g* is the number of generations to the most recent common ancestor for homologous copies identical by descent (Kardos, Qvarnström, and Ellegren 2017; Thompson 2013). We used chromosome‐level mean recombination rates from the *Ficedula* flycatchers to convert the physical lengths of ROH to genetic lengths (cM) and solved for *g* (Kawakami et al. 2014). For ROH on chromosomes > 100 Mb, 50–100 Mb, 25–50 Mb, and 10–25 Mb in size, we used a mean recombination rate of 1.6 cM/Mb, 2.0 cM/Mb, 1.7 cM/Mb, and 1.5 cM/Mb, respectively (Kawakami et al. 2014). Because no mean recombination rates were reported for chromosomes < 10 Mb, we assumed the same recombination rate of 1.5 cM/Mb for ROH located on chromosomes < 10 Mb. We then scaled the number of generations (*g*) to MRCA to years by using a warbler generation time of 2 years (Toews et al. 2016). We also calculated an approximate timeline by subtracting years to MRCA from the sampling year in 2006.

### 2.5 Mutational Classification and Functional Impact

To compare how population demography affects masked and realized load in each of our study species, we used SIFT4G (Sorting Intolerant From Tolerant). SIFT4G uses sequence conservation scores and amino acid properties—to annotate mutation consequences (synonymous, nonsynonymous, stop‐loss, stop‐gain, start‐lost) as well as to predict how those mutations affect protein function (i.e., a “deleterious” effect or a “tolerated” effect) (Vaser et al. 2016). Since no SIFT genomic database exists for *Setophaga*, we custom built one using the Myrtle warbler mywagenomev2.1 assembly (Baiz et al. 2021) following manual instructions (https://github.com/pauline‐ng/SIFT4G_Create_Genomic_DB/tree/master). After building our database, we used the SIFT4G Annotator to annotate our variant list. This generated a vcf‐like dataset containing SIFT4G mutation consequences (synonymous, nonsynonymous, loss of function) and their associated predicted effects (deleterious or tolerated). We removed any sites that were noncoding, were not annotated with a consequence, or in which SIFT failed to produce a prediction. Some sites had multiple mutation consequences and predicted effects assigned to them, a result of multiple transcripts overlapping the region. Sites with multiple mutation consequence categories were only counted once and were counted with the “worst” outcome according to the following hierarchy: loss of function (i.e., stop‐loss, stop‐gain, start‐lost) > nonsynonymous > synonymous. Similarly, for the predicted effects, sites with one or more deleterious predictions were counted as deleterious, whereas tolerated sites were only counted as tolerated if all isoforms were predicted to have tolerated effects. We then merged the resulting SIFT annotations with unpolarized genotype data to count the alternate homozygotes and heterozygotes of each mutation consequence and predicted effect category.

To examine genetic variation unique to each species, we calculated the site frequency spectra for each mutation type, considering only sites with private alternate alleles. Here, we define private alternate alleles as sites where an alternate allele is found exclusively in one or more samples of only one species. After extracting private alternate sites, we subset sites that had SIFT annotations. Using easySFS (Gutenkunst et al. 2009), we calculated the unfolded SFS for each mutation type with a projection of 10, 14, and 14 for *S. citrina, S. ruticilla, and S. kirtlandii*, respectively. We assumed the private alternate allele to be the derived allele since all three species were aligned to an outgroup.

### 2.6 Demographic History

To understand the role of ancient population history on inbreeding and patterns of deleterious variation, we used pairwise sequentially Markovian coalescent (PSMC) V0.6.5‐r67. PSMC uses the density of heterozygotes in 100‐bp windows to infer the time to the most recent common ancestor (Li and Durbin 2011). This method is based on coalescent theory, which indicates that the density of homozygotes and heterozygotes corresponds to the timing of coalescence, and the rate of coalescence is proportional to Ne (Li and Durbin 2011). PSMC requires a consensus diploid sequence for each sample. To create the consensus sequences, we first used the mpileup command in BCFTOOLS on BAM files while filtering for mapping quality (−C 50). The output was piped into the “call” command in BCFTOOLS with the “‐c ‐O v” flags. This produced variant calls that were subsequently converted to fastq using the BCFTOOLS script vcfutils and vcf2fq command. During this step, we also filtered out bases that had mapping quality < 25 (−Q 25) and a minimum read depth of 18 (−d 18). These settings worked well for all but two samples with lower coverage, which we subsequently dropped from this analysis. For the remaining samples, we used fq2psmcfa to convert to the PSMC input format while filtering out any bases with quality < 20 (−q 20). We ran PSMC using settings from Nadachowska‐Brzyska et al. 2016: we ran the program for 30 iterations (−N 30) and set the upper limit of the TMRCA to 5 (−t 5). The ratio of the scaled mutation rate and recombination rate (*q*/*r*) was set to 1 using the ‐r flag (−r 1). We specified Ne to be inferred across 34 free atomic time intervals with the ‐p flag (−p “4 + 30*2 + 4 + 6 +10”). To check for variances in Ne, we performed bootstraps on each sample. We used PSMC's built‐in “splitfa” function to split the consensus sequences into regions, then ran it on 100 randomly selected regions with replacement using the same settings (−N 30, −t 5, −r 1 −p “4 + 30*2 + 4 + 6 + 10”). Plotting was generated in R v4.3.1 with ggplot2 using the plotPsmc R function (Liu and Hansen 2017) and modified scripts from de Jager et al. (2021).

## 3 Results

### 3.1 Sequencing and Variant Calling

Sequencing resulted in moderate average coverage across all individuals: 17X, 18X, and 17X for *S. ruticilla* , *S. kirtlandii* , and *S. citrina* , respectively (Table S1). After calling and filtering variants, our joint vcf consisted of a total of 50,701,237 sites, which included both polymorphic and monomorphic sites. Of these total sites, 18,435,543 were polymorphic in *S. citrina* , 25,204,740 in *S. ruticilla* , and 9,243,730 in *S. kirtlandii* (Table S2). The large number of sites is likely due to aligning all reads to the Myrtle warbler reference, a closely related but distinct species within the same genus. For all our analyses, we treated each sample independently and we found no evidence of admixture between species (Figure S2A). Additionally, our samples did not show within‐species population structure (Figure S2B–D), nor evidence of close familial relationships between individuals (Figure S3).

### 3.2 Runs of Homozygosity

**FIGURE 1:**
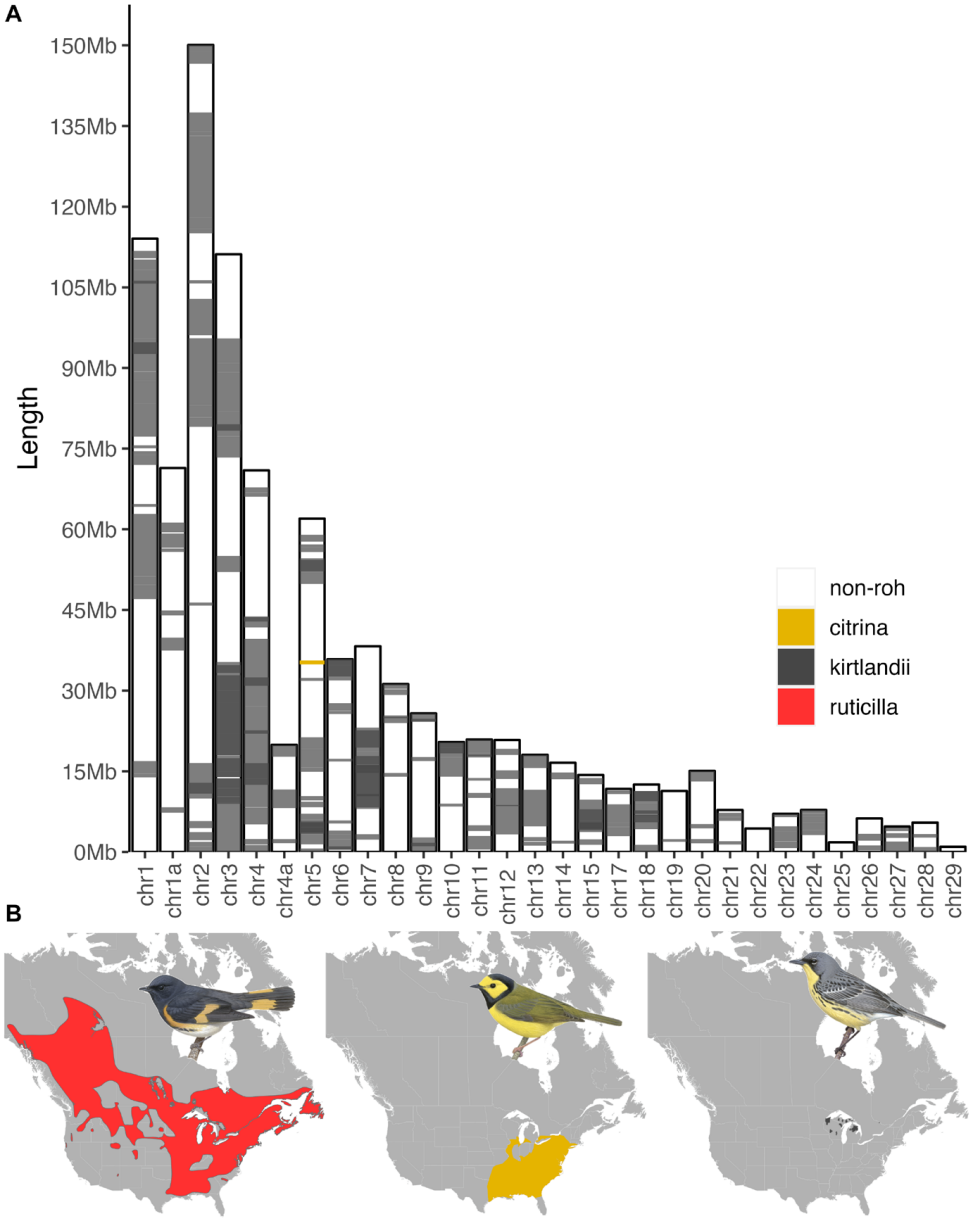
(A) Karyotype with overlapping ROH regions across all samples shows many ROH in *Setophaga kirtlandii* but nearly none in their close relatives. The darker shading indicates regions where ROH from different individuals overlap, and white areas represent non‐ROH regions. (B) Breeding range maps of the American Redstarts ( *Setophaga ruticilla* , Nc = 42,000,000), Hooded Warblers ( *Setophaga citrina* , Nc = 5,200,000), and the Kirtland's warblers ( *Setophaga kirtlandii* , Nc = 4500‐5000) (Partners in Flight 2020).

**FIGURE 2:**
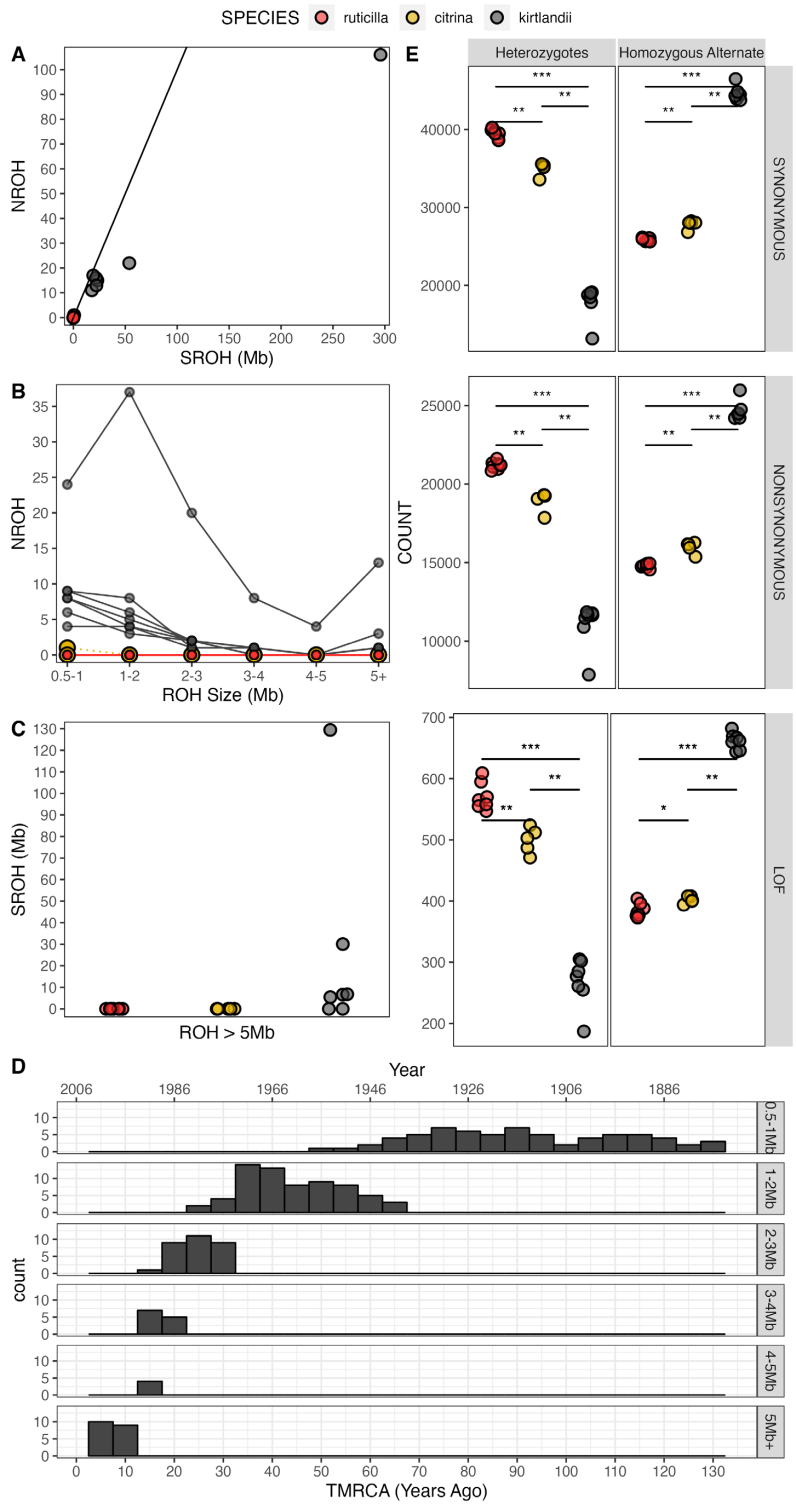
Time to the most recent common ancestor (TMRCA) for ROH haplotypes, and genetic load dynamics shows recent inbreeding and elevated counts of putative deleterious mutations in *S. kirtlandii* . (A) The sum of all ROH lengths (SROH) and number of ROH (NROH) > 0.5 Mb plotted in each species (the *x* = *y* line is for orientation purposes) where each point represents an individual. (B) The number ROH (NROH) plotted for each ROH size category where each line represents an individual. In panels A and B, *S. citrina* and *S. ruticilla* show nearly identical distribution of ROH such that points and lines stack on each other at the axis. Panel (C) shows the total sum of ROH > 5 Mb for each sample where each point represents an individual. (D) Time to the most recent common ancestor (TMRCA) of haplotypes underlying ROH in *S. kirtlandii* samples was aged using estimated recombination rates (cM/Mb) from the *Ficedula* flycatchers and a generation time of 2 years. The secondary axis was scaled by subtracting TMRCA from sample years. (E) The number of genotypes with an alternate allele at synonymous, nonsynonymous, or loss‐of‐function site. In panel E, counts include both polymorphic and monomorphic sites; points jittered to minimize overlap between samples. *p‐value < 0.05, **p‐value < 0.01, ***p‐value < 0.001 by the Mann–Whitney *U* test.

We describe the levels of inbreeding using GARLIC, a model‐based approach that estimates the likelihood a given window is within a run of homozygosity. We categorized ROH into six different size classes, where their lengths reflect the timing of inheritance of identical by descent haplotypes. For example, ROH > 5 Mb represent exceptionally long ROH indicative of recent inbreeding, while ROH < 1 Mb indicate more distant parental relatedness. We found little‐to‐no ROH in either *S. citrina* or *S. ruticilla* —species with large census population sizes and no evidence of recent bottlenecks—while all *S. kirtlandii* had multiple ROH in size categories < 1 Mb, 1–2 Mb, 2–3 Mb, and 3–4 Mb (Figures 1 and 2A,B). These patterns of ROH are consistent with populations that have experienced bottlenecks (Ceballos et al. 2018). We found only one sample that had ROH in the 4–5‐Mb category ( *S. kirtlandii* 183195332), but several *S. kirtlandii* samples had at least one ROH longer than 5 Mb (Figure 2B). *S. kirtlandii* 183195332 alone had 13 ROH in the > 5 Mb category that added up to a total of 129 Mb in ROH, with the longest segment measuring 18.8 Mb (Figure 2C). Taking the proportion of the total ROH relative to the autosomal genome yields the commonly reported inbreeding coefficient, *F*_ROH_. While all *S. ruticilla* samples had no fraction of their genome within ROH (*F*_ROH_ = 0.000), two *S. citrina* samples had *F*_ROH_ = 0.00078 and *F*_ROH_ = 0.00061 and *S. kirtlandii* *F*_ROH_ ranged between 0.0189 and 0.315. Together, these results strongly support recent inbreeding within *S. kirtlandii* .

To understand how the extent of inbreeding changed over time in *S. kirtlandii* , we estimated the number of generations to MRCA (*g*) for haplotypes underlying ROH. Overall, underlying haplotypes in ROH date back to 2–132 years ago from the sampling date in 2006, roughly between the years of 1873–2003 (Figure 2D). Thirty‐four percent of haplotypes underlie ROH 0.5–1 Mb and date back to 52–132 years ago. Thirty‐three percent of ROH were 1–2 Mb and had haplotypes that originated ~25–66 years ago, and 15% of ROH were 2–3 Mb with haplotypes that originated ~17–31 years ago. The remainder underlie ROH > 3 Mb (16%) and arose between ~2.6–20 years ago from the sampling date (Figure 2D, Table S3). Taken together, this signifies haplotypes underlying ROH > 1 Mb originated during or shortly after the bottleneck in the 1950s. But given that some of these haplotypes originated before the 1950s indicates that the bottleneck probably happened a decade earlier, when formal census counts were not yet conducted. The presence of young haplotypes indicates that close inbreeding was happening even while the population was recovering.

### 3.3 Deleterious Variation and Risk of Inbreeding Depression

To investigate whether *S. kirtlandii* has an increased risk of inbreeding depression, we explored the distribution of deleterious variation. Using predicted gene annotations from our reference genome, we used SIFT4G to annotate the mutation consequences of all coding variants as well as their predicted impact on protein function. SIFT annotated 549,797 mutations, of which 337,136 were synonymous, 207,443 were nonsynonymous, and 5218 were loss of function (4088 stop‐gain, 280 stop‐loss, 850 start‐lost). More synonymous sites were observed across all species compared to nonsynonymous sites. Likewise, across species, there were more nonsynonymous sites than loss‐of‐function sites. As expected for species with large populations, *S. ruticilla* and *S. citrina*, most mutations are heterozygotes. By contrast, *S. kirtlandii* had most mutations as homozygotes consistent with their higher *F*_ROH_ values (Figure 2E).

**FIGURE 3:**
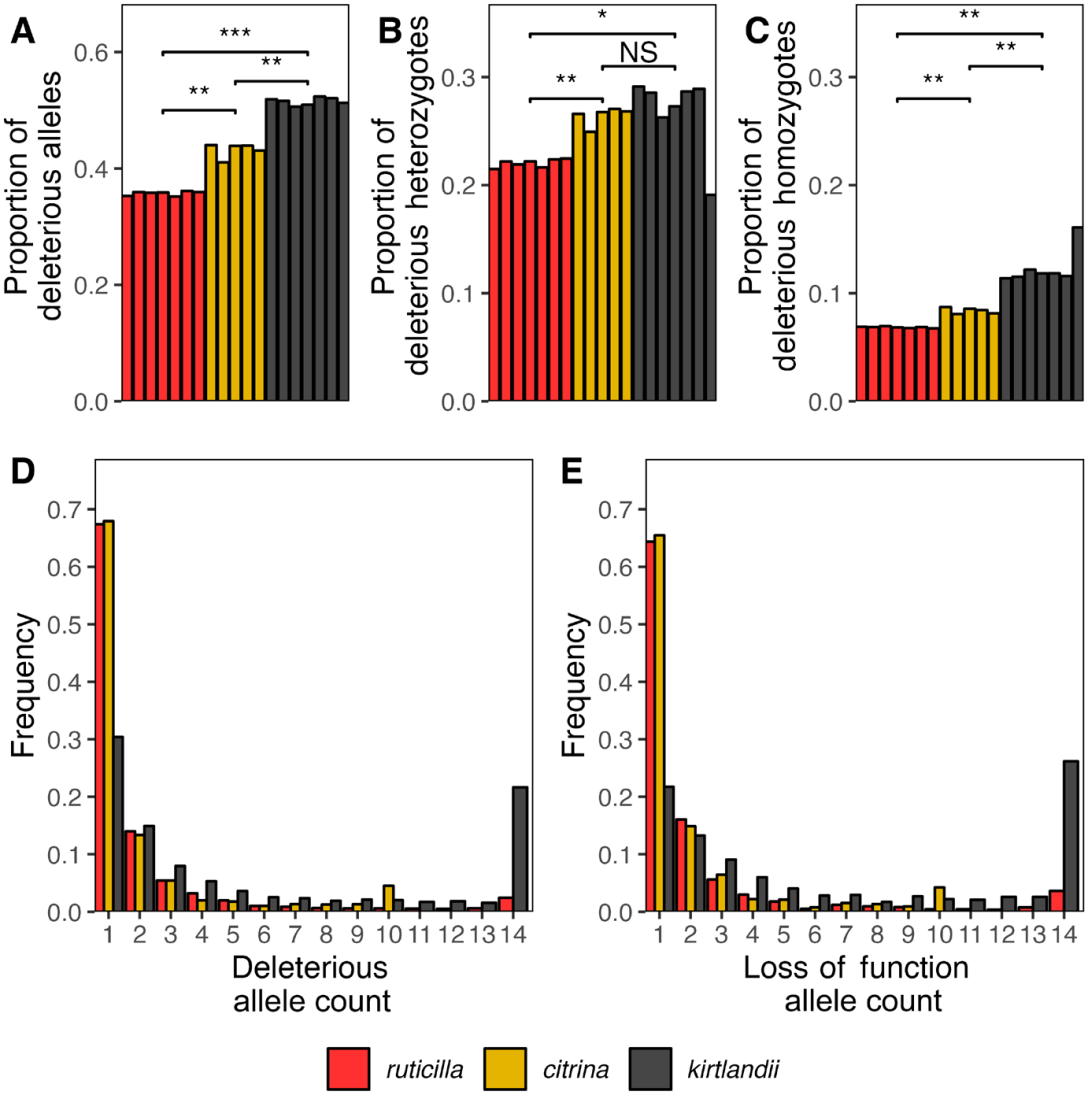
(A) Proportion of alternate alleles at polymorphic sites predicted to be deleterious. (B) Proportion of heterozygotes at polymorphic sites that are predicted to be deleterious. (C) Proportion of alternate homozygotes at polymorphic sites predicted to be deleterious. NS, Not Significant, *p‐value < 0.05, **p‐value < 0.01, ***p‐value < 0.001 by the Mann–Whitney *U* test. Unfolded site frequency spectra for sites where private alternate alleles are predicted to be (D) deleterious and (E) loss of function. In both panels D and E, the frequency is calculated as the number of private alternate alleles deleterious or loss of function relative to the total number of private alternate alleles of each mutation type.

Next, we consider the SIFT prediction effects on protein function. Our SIFT database consisted of 536,219 predicted effects. It is important to note that our predicted effect database has fewer sites than mutations because either loss‐of‐function mutations do not get a prediction effect or the prediction effect of the mutation was unknown. Of the total sites that did get a prediction effect, 15.9% of sites were predicted to be deleterious and 84% were predicted to be tolerated. We investigated how deleterious variation is distributed across each species by calculating the proportion of alternate‐allele homozygotes and heterozygotes in each species. We find that the higher proportion of alternate sites predicted to be deleterious seems to be associated with population size; *S. ruticilla* with the largest population size having the least, followed by *S. citrina* and *S. kirtlandii* (Figure 3A, Table S4). While most deleterious sites occur in heterozygosity for all species (Figure 3B), *S. kirtlandii* had the highest proportion of deleterious heterozygotes overall. Although this difference was significant when compared to *S. ruticilla* (*p*‐value = 0.02622 by the Mann–Whitney *U* Test), it was not significantly different from *S. citrina* (Figure 3B; *p*‐value = 0.202 by the Mann–Whitney *U* Test). While large populations can maintain their genetic load in heterozygosity, also known as “masked load”, in small populations, this masked load may become realized with increased inbreeding. *S. kirtlandii* has a significantly higher proportion of alternate deleterious homozygotes when compared to *S. citrina* and *S. ruticilla* (*p*‐value = 0.002525, *p*‐value = 0.002141, respectively, by the Mann–Whitney *U* Test) (Figure 3C). This indicates that in addition to their higher masked load, they also have a higher realized load.

We next considered the distribution of frequencies of mutations of various classes (synonymous, nonsynonymous, loss of function, noncoding, deleterious, and tolerated) unique to each species. In other words, we considered mutations only if they were found at sites with a private alternate allele. We assume the private alternate allele to be the derived allele since all samples were aligned to an outgroup. Unfolded site frequency spectra show that the allele frequencies of each mutation class differ across species (Figure S4A–C). Regardless of mutation class, *S. kirtlandii* has a higher proportion of high frequency and fixed mutations at purported derived sites (Figure S4A–C). This result reflects the reduction in genetic diversity that is characteristic of small, bottlenecked populations, but it is of particular concern for deleterious sites (Figure 3D) and loss‐of‐function mutations (Figure 3E), which may negatively impact fitness.

### 3.4 Demographic History

**FIGURE 4:**
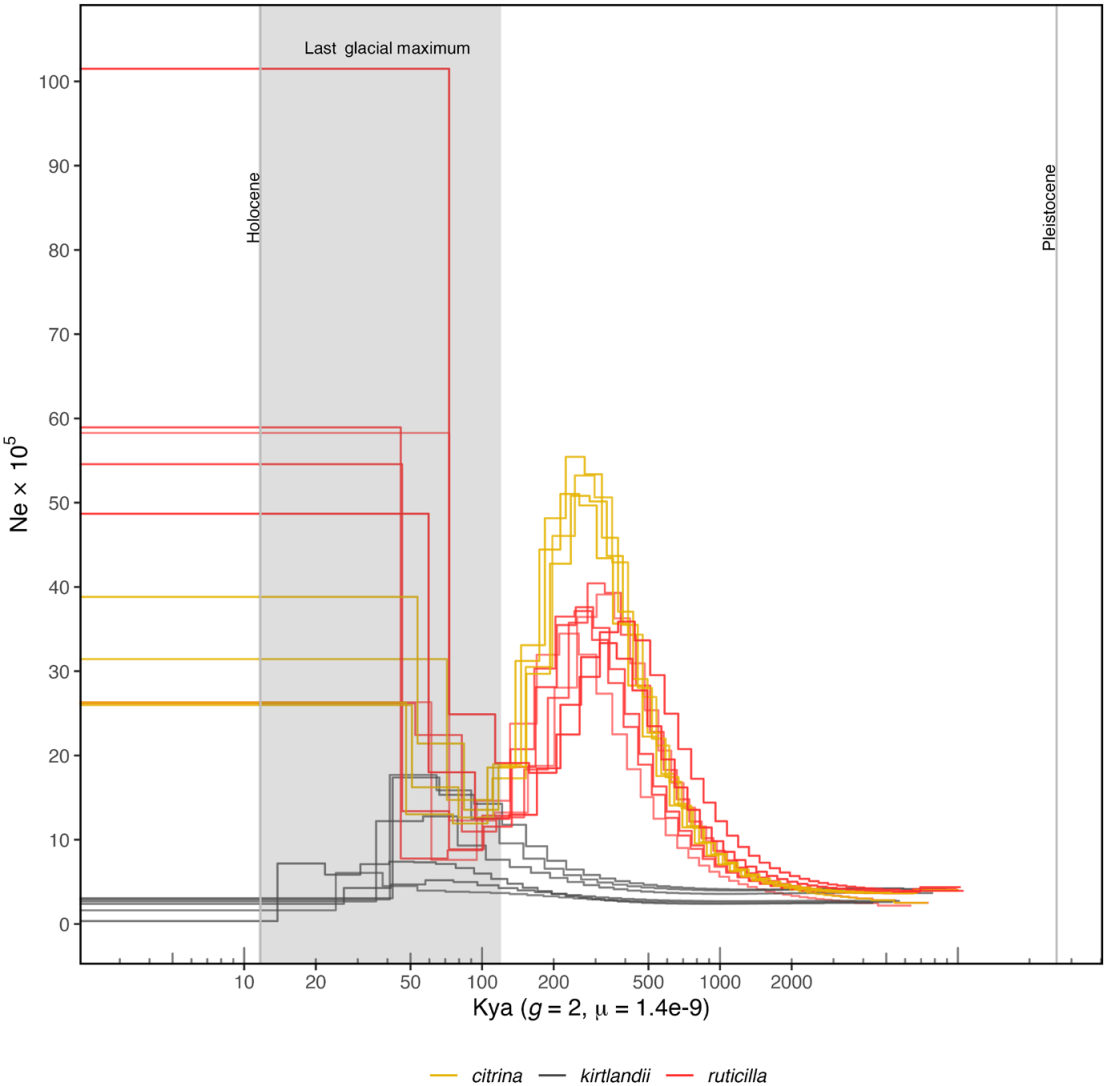
Temporal dynamics of effective population size using pairwise sequentially Markovian coalescent (PSMC). The *x*‐axis is thousands of years before present (Kya) and has been calibrated using a generation time (*g*) of 2 years and a per‐site mutation rate (μ) of 1.4e‐9.

We conducted PSMC analysis to explore the role of ancient demographic history on patterns of inbreeding and deleterious variation (Figure 4; and Figure S5). First, our analysis suggests an event of population divergence in the last common ancestor, which may have resulted in the speciation of *S. kirtlandii* during the Pleistocene, possibly as the last common ancestor expanded its range. Following population divergence, PSMC analysis shows different trajectories for each species. Steady population expansion started around ~1.5 Mya, albeit with *S. kirtlandii* populations on a slower rate of expansion. Population trajectories also suggest that a second population divergence event occurred roughly 400 Kya. Shortly after populations diverged, *S. citrina* and *S. ruticilla* populations expanded and contracted during the onset of the last glacial maximum (LGM), while *S. kirtlandii* populations increased slowly into the mid‐LGM before contracting as well. In the late LGM and early Holocene, only *S. citrina* and *S. ruticilla* populations expanded and remained large and stable to the present day.

## 4 Discussion

With highly inbred populations that lack strong population structure, whole‐genome comparisons between closely related species with different population histories can be used to explore the genomic consequences of demographic processes like strong bottlenecks. Specifically, our analysis shows many small‐ to medium‐sized ROH segments in all Kirtland's warblers but nearly zero ROH in any of their closest relatives, American Redstarts and Hooded Warblers. The discovery that Kirtland's warblers contain exceptionally long runs of homozygosity indicates that these individuals are the product of very recent inbreeding events, which had not previously been examined in the context of recovery planning for this important songbird species.

We found that the highest portion (~34%) of all ROH in our samples were very short segments of identical‐by‐descent haplotypes that date between 1874 and 1954. This indicates that most haplotypes underlying small ROH originated prior to any known bottlenecks and could be the result of persistently small ancestral population sizes, which we demonstrate here for the first time using PSMC. Despite presumably ample habitat availability in jack pine forest during the last glacial maximum (Godbout et al. 2005), Kirtland's warblers' slow rate of population expansion following its divergence could indicate that their habitat specificity in early successional jack pine habitats is an ancient evolutionary adaptation and could account for long‐term small effective population sizes compared to its close relatives (Tucker et al. 2016). ROH between 1 and 2 Mb, on the other hand, accounts for the second highest portion of all ROH with haplotypes that date between 1940 and 1981. This is consistent with a strong and sudden population collapse beginning in the early 1940s. Although there are no formal census counts to confirm this and our PSMC analysis lacks enough resolution to estimate recent population trajectories, Wilson, Marra, and Fleischer (2012) report a drastic population decrease attributed to the suppression of large‐scale wildfires during 1946–1980. Molecular estimates of effective population sizes also indicate a decrease from Ne = 259 pre‐bottleneck to Ne = 161 post‐bottleneck (Wilson, Marra, and Fleischer 2012), which supports a rapid decline. Although conservation efforts started in the 1960s, Kirtland's warbler populations stalled and only began to recover until the early 1990s. Despite this recovery, the longest ROH comprises the youngest haplotypes estimated to have originated 2–12 years prior to the sampling date in 2006 and indicates that close inbreeding occurred at the time of population recovery.

While estimates of TMRCA for haplotypes underlying ROH align well with population surveys, it is also important to consider that recombination rates vary across the genome and can affect our TMRCA estimates. For example, haplotypes located in low recombinant regions would increase TMRCA as they would persist for longer, while those located in high recombination regions are broken down faster and decrease TMRCA. For this analysis, we used chromosome‐level mean recombination rates from *Ficedula* flycatchers as we have yet to characterize the recombination landscape for parulid warblers. That said, using a mean recombination rate of 1.5 cM/Mb from a highly divergent species, like the Zebra Finch ( *Taeniopygia guttata* ), would still only increase estimated TMRCA by a few years (Backström et al. 2010). Although recombination maps are not yet available for parulids, understanding how genome‐wide recombination affects the persistence of long haplotypes and the deleterious variation harbored therein would help predict the fate of deleterious variation.

Our analysis shows that Kirtland's warblers have significantly more nonsynonymous and loss‐of‐function alternate homozygotes. The mutation consequences alone, however, tells us very little about the impacts on protein function, which taken together could influence individual fitness. Due to the flexibility in protein coding sequences, amino acids may sometimes be interchangeable as they have compatible biochemical properties, such that not all nonsynonymous mutations are deleterious. To this end, we showed that Kirtland's warblers also have a higher proportion of deleterious alternate homozygotes. At the population level, this translates to a higher proportion of fixed derived deleterious variation. These patterns of genetic variation are consistent with theoretical expectations, which predict that strong genetic drift increases the fixation of deleterious alleles (Robinson et al. 2023). While our samples may not be representative of the entire population, our data raise concerning and unexpected inferences about the number of fixed variants among Kirtland's warblers.

The effect of bottlenecks on genetic variation is not straightforward because while they can reduce genetic load (Grossen et al. 2020; Leon‐Apodaca et al. 2024; Robinson et al. 2018; Xue et al. 2015), they can also increase genetic load by increasing the frequency of mildly deleterious variants due to drift (Mathur and DeWoody 2021; Robinson et al. 2023; Steux and Szpiech 2024). Relaxed natural selection on mildly damaging recessive variants could explain the higher proportion of deleterious homozygotes. When focusing on private mutations, we find that Kirtland's warblers have proportionally more high‐frequency deleterious alleles (Figure S4). This means that the bottleneck and inbreeding may have not led to purging in Kirtland's warblers despite their long‐term small Ne, but it rather played a larger role in increasing the frequencies of deleterious variation by redistributing alleles into homozygous form. While the higher fixation of deleterious variation in Kirtland's warblers could point to higher genetic load, it is inherently difficult to assess without information on the distribution of fitness effects, dominance coefficients, and their interactions. In bottlenecked populations, common variants are predicted to account for a larger percentage of genetic load (Lohmueller 2014a, 2014b). Theoretical models on the impact of demography on genetic architecture in humans suggest that common variants, which are likely to be weakly selected and recessive, contribute considerably less to the variance of complex traits (e.g., disease) (Simons et al. 2014). Given their long‐term small populations, it is possible that the deleterious alleles found in Kirtland's warblers are likely weak and less recessive. However, this does not mean that Kirtland's warblers are not at risk of inbreeding depression as the role of accumulated weakly deleterious mutations on the probability of extinction, known as mutational meltdown, remains uncertain (Robinson et al. 2023).

To our knowledge, there is no evidence that Kirtland's warblers suffer from visible phenotypic abnormalities. These kinds of abnormalities have been observed in the highly inbred Isle Royale wolves, including cases of syndactyly, malformed vertebrate, and rope‐tail (Robinson et al. 2019). In our sample of Kirtland's warblers, the individual with the highest ROH load had unremarkable morphometric and plumage traits, although we do not know about its reproductive success. In other species, inbreeding depression only became apparent after an environmental challenge (Keller et al. 1994) or as a longer‐term reduction in reproductive fitness. For instance, in the critically endangered New Zealand Kakapō ( *Strigops habroptilus* ), studies have found low sperm quality and in the endangered hihi ( *Notiomystis cincta* ) they found smaller clutch sizes, sex‐biased mortality, and lower juvenile survival (Brekke et al. 2010; Duntsch et al. 2023; Dussex et al. 2021). Quantifying the effect of mutations on reproductive fitness in Kirtland's warblers will be substantially more difficult because their reproductive success has also been heavily impacted by brood parasitism. Disentangling the effect of ecological versus genetic factors, or a combination, on population dynamics and fitness is a challenge that will be explored by future work.

As of 2019, Kirtland's warblers are no longer federally protected (Fish and Wildlife Service 2019). It is impossible to know, given these new results of substantial inbreeding, how this knowledge might have figured into the delisting process. Either way, this serves as an important case study to show that while population growth plays a key role in population's recovery, exploring the underlying genomic substrate may reveal important conservation considerations that population numbers cannot (Femerling et al. 2023; Feng et al. 2019). Additionally, previous studies reporting a slight loss of genetic variation and unaffected heterozygosity levels were used as a lack of evidence for inbreeding in the delisting of the Kirtland's warblers. This case further highlights that although simple metrics of diversity may not fully encapsulate the complicated genetic patterns of population trajectories as others have pointed out (Teixeira and Huber 2021), they may point to a more nuanced indication of eroding genetic diversity and should be considered carefully when making important conservation decisions (Exposito‐Alonso et al. 2022; Kardos et al. 2021; Laikre et al. 2020). While they are celebrated as a quintessential conservation success story, Kirtland's warblers are highly inbred and have a high frequency of fixed deleterious variants that can quietly impact their ability to persist. Moving forward, however, we also see that this is also an opportunity to study how severely bottlenecked populations might recover genetic diversity following periods of intense conservation.

## Conflicts of Interest

The authors declare no conflicts of interest.

## Supporting information


Appendix S1.

## Data Availability

Raw sequence data of each of the newly sequenced *Setophaga* genomes generated in this study are available on SRA under project PRJNA630247. Code used to perform the analysis presented in this study is available on GitHub https://github.com/calderonanna/Warbler‐ROH.
