# Supplementary material for: Recently Delisted Songbird Harbors Extensive Genomic Evidence of Inbreeding, Potentially Complicating Future Recovery
Source: Evol Appl. 2024 Dec 9;17(12):e70052. doi: 10.1111/eva.70052 (PMC11627117; doi:10.1111/eva.70052)
Supplement: Supplementary file 1 — Appendix S1. [file EVA-17-e70052-s001.docx]

SUPPLEMENTARY MATERIAL

​​**Table S1:** Samples were obtained through various museums and institutes and were collected between 2003-2020. **Indicates samples from a previous study that were re-sequenced to achieve a higher coverage. *New samples were only sequenced once to mid-coverage.

| **Collection** | **Species** | **Sample** | **Source** | **Sex** | **Prep** | **Date** | **Alignment %** | **Cov** | **Accession** |
| --- | --- | --- | --- | --- | --- | --- | --- | --- | --- |
| CUMV | ruticilla | 163 | Baiz et al.** | M | Tissue | 2003-06-26 | 87.33 | 15.49 | SAMN16870856 |
| CUMV | ruticilla | 1049 | Baiz et al.** | M | Tissue | 2003-07-07 | 85.98 | 17.69 | SAMN16870855 |
| CUMV | ruticilla | 1940 | Baiz et al.** | M | Tissue | 2007-05-15 | 86.56 | 18.28 | SAMN16870857 |
| CUMV | ruticilla | 4056 | Baiz et al.** | M | Tissue | 2013-05-17 | 87.83 | 17.20 | SAMN16870858 |
| D.Toews | ruticilla | 284029323 | Baiz et al.** | M | Blood | 2017-06-09 | 87.86 | 16.53 | SAMN16870853 |
| D.Toews | ruticilla | 283030179 | New* | M | Blood | 2020-06-19 | 88.59 | 18.17 | SAMN40160019 |
| D.Toews | ruticilla | 283030100 | New* | M | Blood | 2020-06-03 | 87.99 | 19.61 | SAMN40160020 |
| CUMV | citrina | 262 | Baiz et al.** | M | Tissue | 2003-08-24 | 84.80 | 18.09 | SAMN16913428 |
| CUMV | citrina | 2871 | Baiz et al.** | M | Tissue | 2009-08-25 | 86.35 | 12.56 | SAMN16913429 |
| D.Toews | citrina | 283030065 | New* | M | Blood | 2020-05-22 | 86.43 | 18.67 | SAMN40160016 |
| D.Toews | citrina | 283030083 | New* | M | Blood | 2020-05-30 | 87.26 | 17.87 | SAMN40160017 |
| D.Toews | citrina | 284029450 | New* | M | Blood | 2019-05-25 | 87.04 | 18.27 | SAMN40160018 |
| Smithsonian | kirtlandii | 183195332 | Baiz et al.** | M | Blood | 2006-06-10 | 86.79 | 15.20 | SAMN16913439 |
| Smithsonian | kirtlandii | 183194861 | Baiz et al.** | M | Blood | 2006-06-07 | 87.28 | 23.05 | SAMN16913440 |
| Smithsonian | kirtlandii | 183195321 | Baiz et al.** | M | Blood | 2006-06-07 | 86.77 | 24.01 | SAMN16913441 |
| Smithsonian | kirtlandii | 183195304 | Baiz et al.** | M | Blood | 2006-05-24 | 85.73 | 14.02 | SAMN16913442 |
| Smithsonian | kirtlandii | 183194841 | Baiz et al.** | M | Blood | 2006-05-27 | 87.27 | 15.06 | SAMN16913443 |
| Smithsonian | kirtlandii | 183195326 | New* | M | Blood | 2006-06-08 | 87.03 | 16.53 | SAMN40160021 |
| Smithsonian | kirtlandii | 183195312 | New* | M | Blood | 2006-05-27 | 87.53 | 20.50 | SAMN40160022 |

**Table S2:** Monomorphic and polymorphic sites were filtered out separately per species.

| **Species** | **Monomorphic Sites** | **Polymorphic Sites** | **Total Sites** |
| --- | --- | --- | --- |
| S. ruticilla | 25496498 | 25204739 | 50701237 |
| S. citrina | 32265694 | 18435543 | 50701237 |
| S. kirtlandii | 41457508 | 9243729 | 50701237 |

**Table S3:** Size distribution of all ROH in *S.kirtlandii*, and the underlying haplotypes' estimated time to the most recent common ancestor from the sampling year in 2006. NROH indicates the number of segments of ROH from all samples, and PROH indicates the proportion of ROH within a certain size.

| **ROH Size (Mb)** | **NROH** | **PROH** | **TMRCA (Years)** | **Time Span (Years)** |
| --- | --- | --- | --- | --- |
| 0.5-1 | 68 | 0.3417085427 | 52-132 | 80 |
| 1-2 | 66 | 0.3316582915 | 25-66 | 41 |
| 2-3 | 30 | 0.1507537688 | 17-31 | 14 |
| 3-4 | 12 | 0.06030150754 | 14-20 | 6 |
| 4-5 | 4 | 0.02010050251 | 13-15 | 2 |
| 5+ | 19 | 0.09547738693 | 2-12 | 10 |

**Table S4**: The proportion of polymorphic sites predicted to be deleterious. Proportions account for all genotypes including reference homozygotes, alternate homozygotes, and heterozygotes

| **Species** | **Sample** | **Proportion** |
| --- | --- | --- |
| S. ruticilla | 163 | 0.1449382 |
|  | 1049 | 0.1478797 |
|  | 1940 | 0.1486886 |
|  | 4056 | 0.1470901 |
|  | 284029323 | 0.1451705 |
|  | TF19T04 | 0.1493388 |
|  | TF03T03 | 0.150139 |
| S. citrina | 262 | 0.1483599 |
|  | 2871 | 0.1393611 |
|  | TE22T01 | 0.1488338 |
|  | TE30T02 | 0.1485093 |
|  | SE25T02 | 0.1485814 |
| S. kirtlandii | 183195332 | 0.1643903 |
|  | 183194861 | 0.1672599 |
|  | 183195321 | 0.1670899 |
|  | 183195304 | 0.1626005 |
|  | 183194841 | 0.1642703 |
|  | 183195326 | 0.16638 |
|  | 183195312 | 0.1691597 |


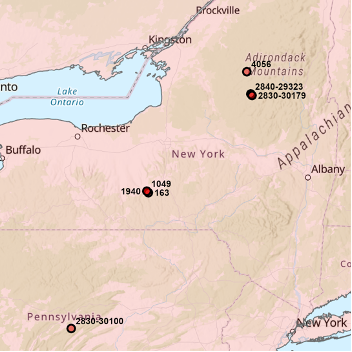

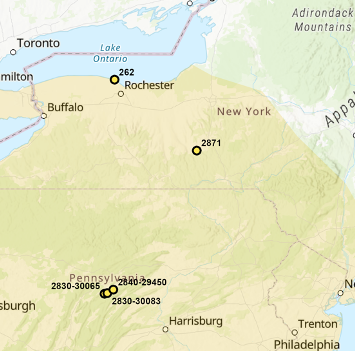

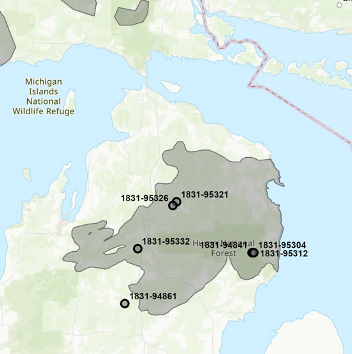


| *S. ruticilla* | *S. citrina* | *S. kirtlandii* |
| --- | --- | --- |

**Figure S1:** Map of all sampling locations in relation to species breeding range. All *S. kirtlandii* samples were taken in 2006 from their central breeding range in the north of Lower Michigan where they exclusively bred.


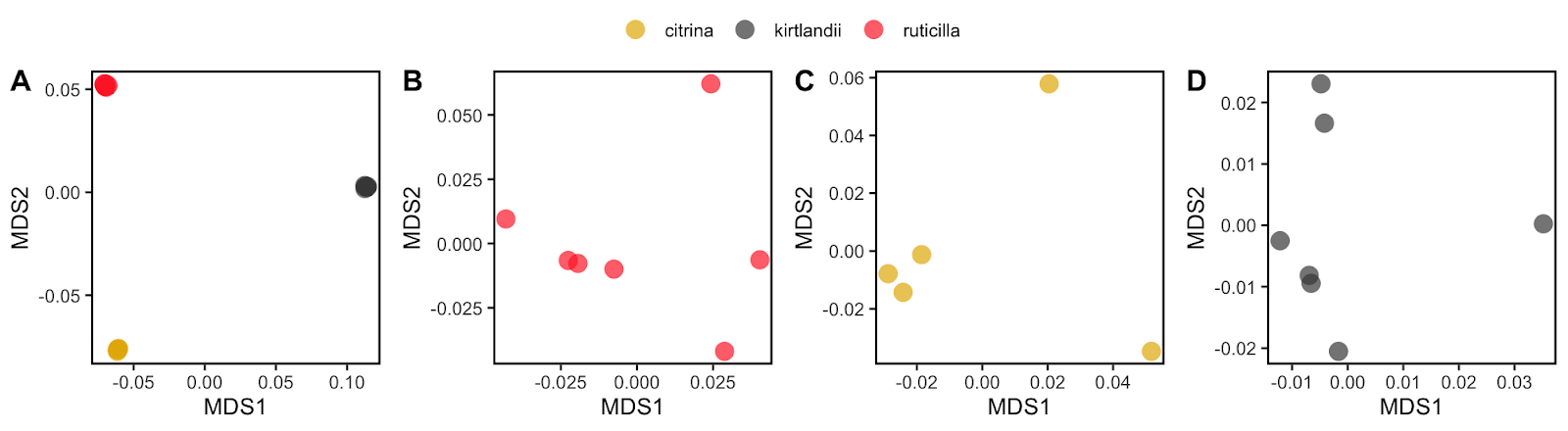


**Figure S2**: Multidimensional scaling analysis (MDS) **(A)** shows no admixture between species, and no population structure within each species **(B-D)**


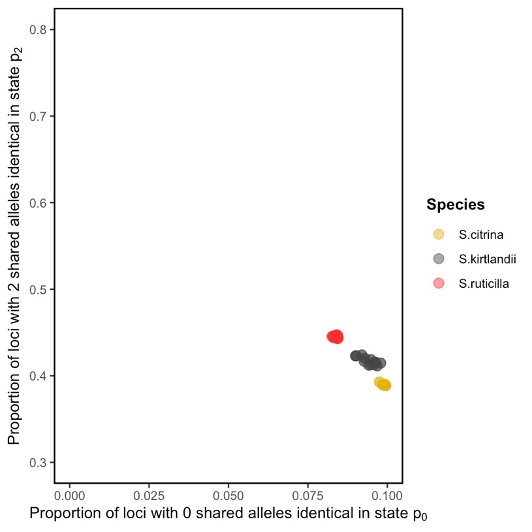


**Figure S3**: Allele sharing analysis shows allele sharing for paired individuals within each species. Interpretation for this analysis is from Pemberton el al. 2010, which describes points clustered in the fourth quadrant (lower right) with low P_0_ and P_2_ values indicating unrelated individuals.


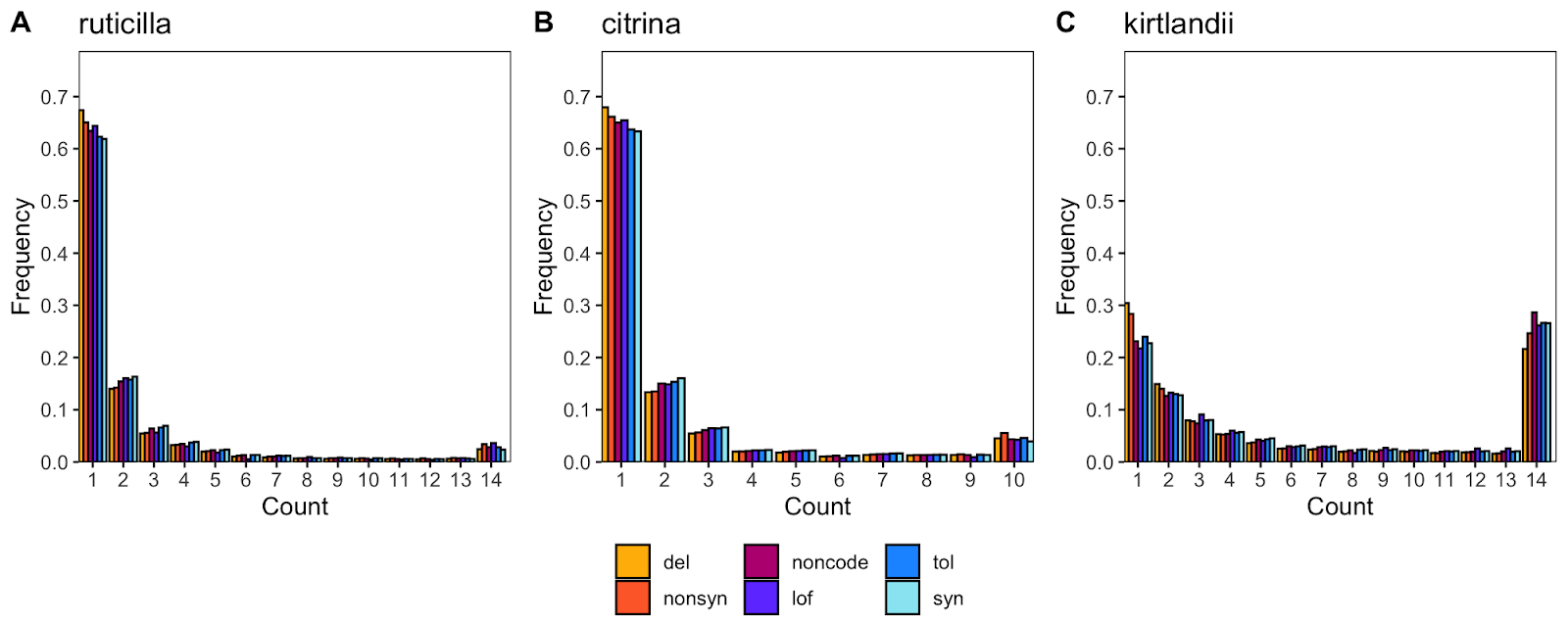


**Figure S4:** Unfolded site frequency spectra for each mutation type with a private alternate allele. SFS is normalized against the total number of private alternate sites of that particular mutation and the private alternate allele is assumed to be the derived allele. SFS shows that while all species have equal proportions of private mutations, their allele frequencies differ across species.

| 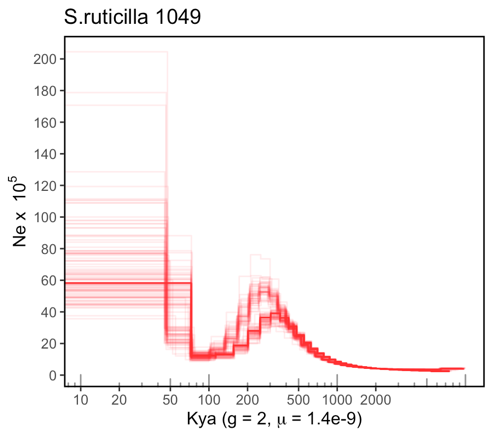 | 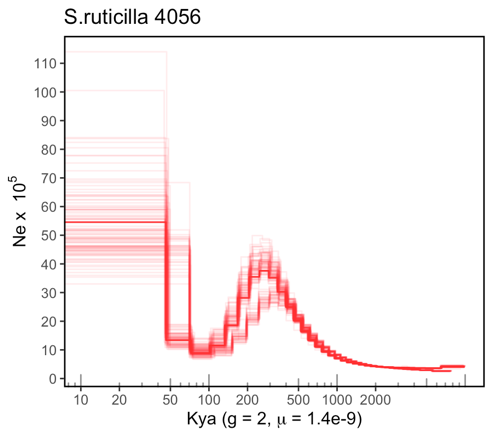 | 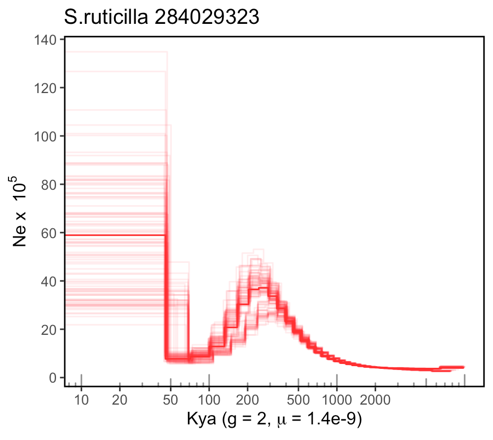 |
| --- | --- | --- |
| 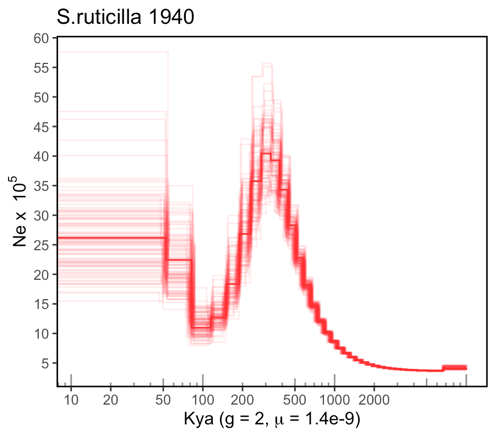 | 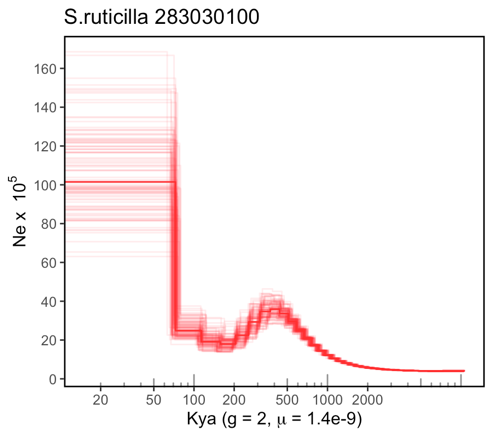 | 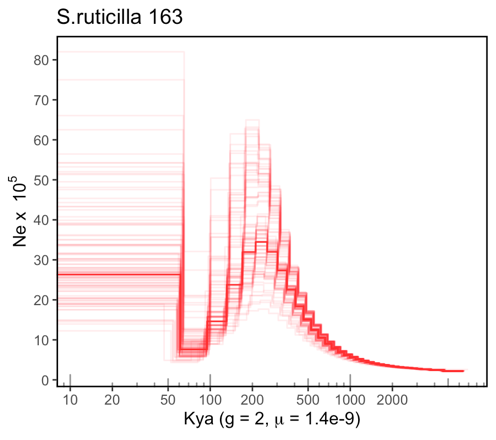 |
| 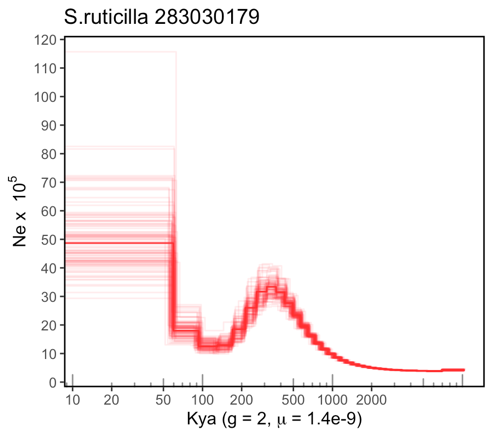 | 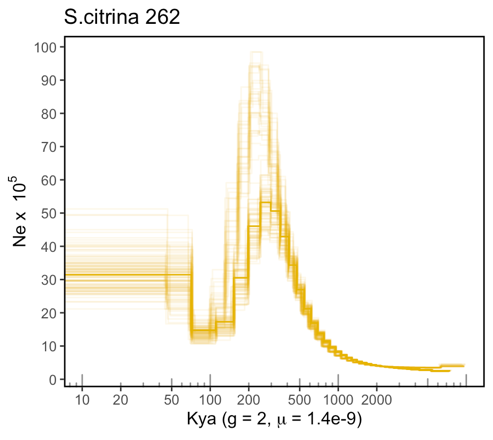 | 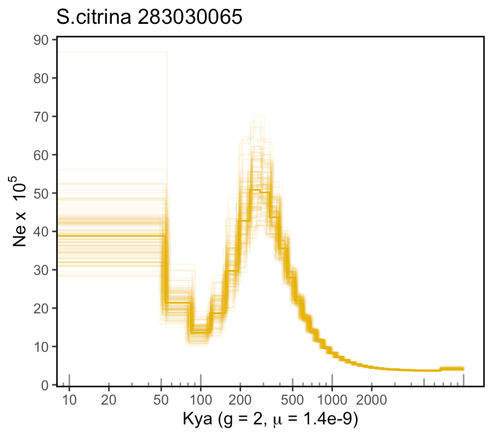 |
| 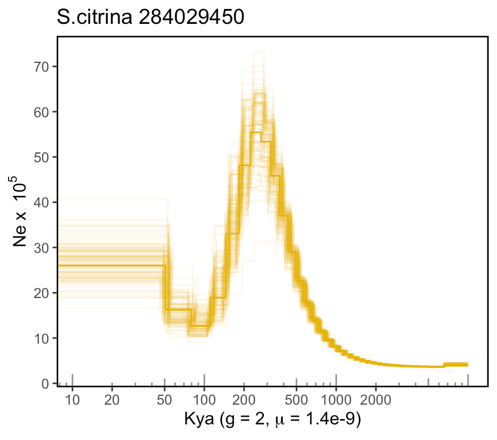 | 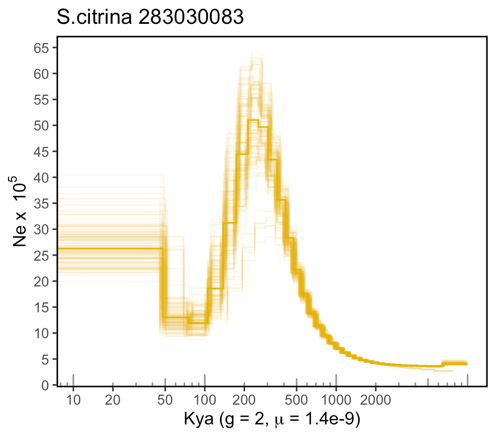 | 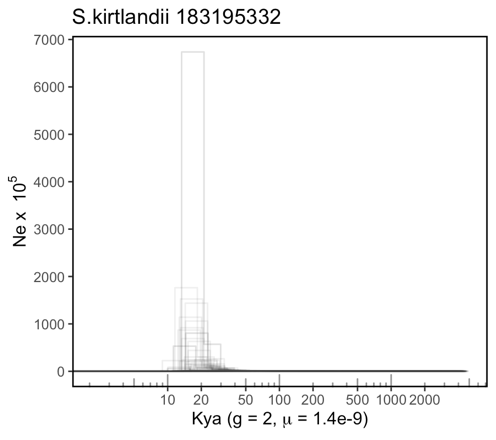 |
| 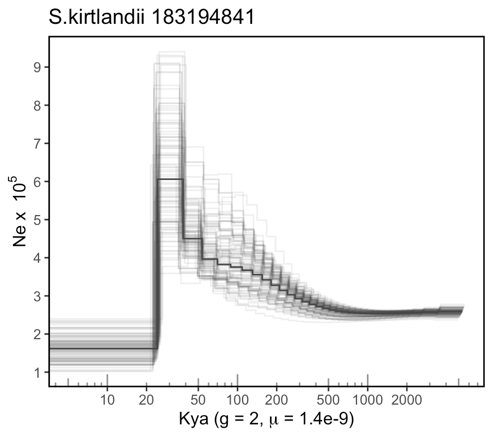 | 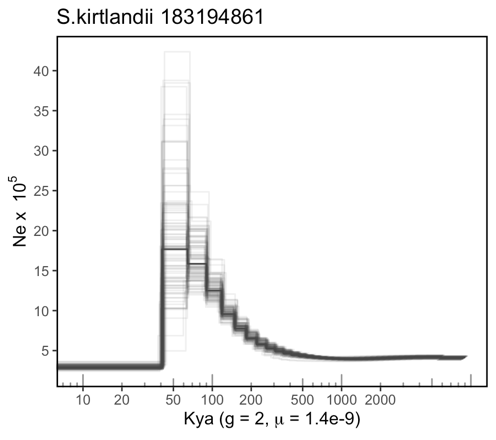 | 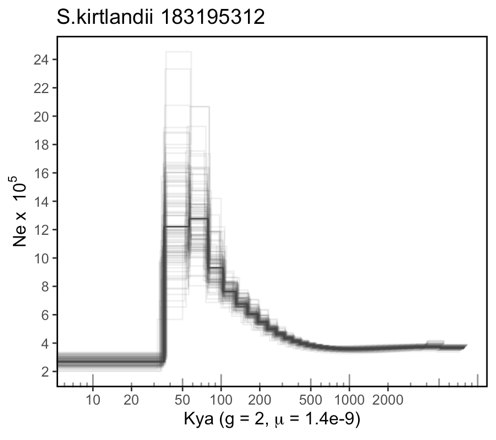 |
| 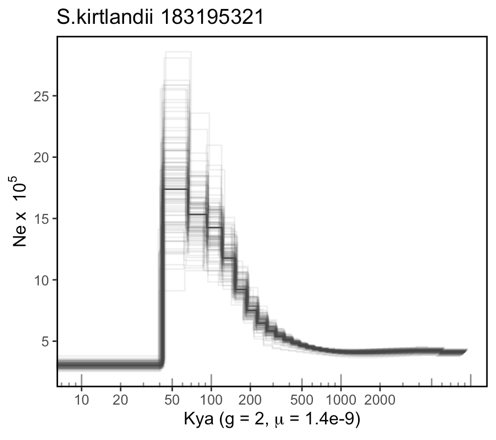 | 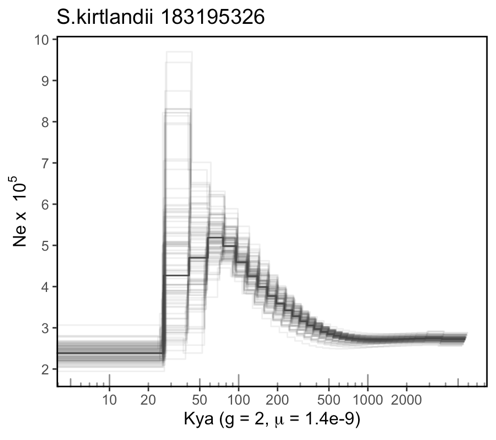 |  |

**Figure S5:** Pairwise sequentially Markovian coalescent (PSMC). Solid line indicates the demographic inference for each sample with 100 bootstrap replicates overlaid as faded lines. The x-axis has been calibrated using a generation time (g) of 2 years and a per site mutation rate (μ) of 1.4e-9.
